# Maternal effects on male weaponry: female dung beetles produce major sons with longer horns when they perceive higher population density

**DOI:** 10.1186/1471-2148-12-118

**Published:** 2012-07-23

**Authors:** Bruno A Buzatto, Joseph L Tomkins, Leigh W Simmons

**Affiliations:** 1 Centre for Evolutionary Biology, School of Animal Biology (M092), The University of Western Australia, 35 Stirling Highway, Crawley, 6009, WA, Australia

**Keywords:** Maternal effects, Population density, Male dimorphism, Polyphenism, Phenotypic plasticity

## Abstract

**Background:**

Maternal effects are environmental influences on the phenotype of one individual that are due to the expression of genes in its mother, and are expected to evolve whenever females are better capable of assessing the environmental conditions that their offspring will experience than the offspring themselves. In the dung beetle *Onthophagus taurus,* conditional male dimorphism is associated with alternative reproductive tactics: majors fight and guard females whereas minors sneak copulations. Furthermore, variation in dung beetle population density has different fitness consequences for each male morph, and theory predicts that higher population density might select for a higher frequency of minors and/or greater expenditure on weaponry in majors. Because adult dung beetles provide offspring with all the nutritional resources for their development, maternal effects strongly influence male phenotype.

**Results:**

Here we tested whether female *O. taurus* are capable of perceiving population density, and responding by changing the phenotype of their offspring. We found that mothers who were reared with other conspecifics in their pre-mating period produced major offspring that had longer horns across a wider range of body sizes than the major offspring of females that were reared in isolation in their pre-mating period. Moreover, our results indicate that this maternal effect on male weaponry does not operate through the amount of dung provided by females to their offspring, but is rather transmitted through egg or brood mass composition. Finally, although theory predicts that females experiencing higher density might produce more minor males, we found no support for this, rather the best fitting models were equivocal as to whether fewer or the same proportions of minors were produced.

**Conclusions:**

Our study describes a new type of maternal effect in dung beetles, which probably allows females to respond to population density adaptively, preparing at least their major offspring for the sexual competition they will face in the future. This new type of maternal effect in dung beetles represents a novel transgenerational response of alternative reproductive tactics to population density.

## Background

Traditionally, maternal effects were understood to occur whenever the mother’s phenotype directly affects the phenotype of their offspring, regardless of the female’s genetic contributions to her offspring [1]. A more recent definition states that maternal effects are environmental influences on the phenotype of one individual that are due to the expression of genes in its mother [2]. Therefore, maternal effects represent developmental influences extended across life cycle stages, in which genetic or environmental differences in the maternal generation are expressed as phenotypic differences in the offspring [3]. Maternal effects are known to act on a multitude of offspring traits, such as body size, condition, and even expression of sexually selected traits [4-6]. Regarding dimorphic traits, maternal effects seem to be especially important for the expression of the “life cycle polymorphisms” (*sensu*[7]) of insects, such as: sexual versus parthenogenic morphs in aphids, winged versus wingless morphs in the red linden bug, summer versus winter morphs in the collembolan *Orchesella cincta*, as well as dispersal versus sedentary morphs in the cowpea seed beetle and in several species of locusts [3]. Maternal effects also control the expression of what Roff [7] called “protective polymorphisms” in species of the crustacean genus *Daphnia*. An elongated structure that grows from behind the head of these animals is a threshold-trait that functions as an anti-predator defense [8], and its expression is influenced by maternal effects [9].

Male dimorphism [10] usually reflects alternative reproductive tactics among males: the large (‘major’) male morph typically has more elaborate weaponry, and guards females or reproductive territories, while the small (‘minor’) male morph has reduced weaponry and sneaks copulations [11]. Male dimorphism is now known to be taxonomically widespread [12], having been described in taxa as distinct as molluscs [13-16], nematodes [17], and predominantly vertebrates and arthropods (reviewed by [12]). Most cases of male dimorphism seem to be due to alternative tactics within a conditional strategy, in which the phenotype expressed by individuals is influenced by their status [11,18,19], but see [20]. According to the conditional strategy individual fitness varies as a function of competitive ability (i.e., status [11]), for the alternative tactics to be evolutionarily stable in the population the rate of change in fitness with status needs to differ between the alternative tactics such that the fitness functions intersect. The exact point at which selection favors a switch from one tactic to another can be calculated from knowledge of the fitness functions, the distribution of switchpoints and the variation in (in this case) status [18,19]. As a result, males with a status higher than such a switchpoint benefit from adopting the major phenotype (the primary tactic), whereas males with status lower than the switchpoint benefit from adopting the minor phenotype (the alternative tactic, [11,18,19]).

In theory, the functions that relate the fitness of each male phenotype to individual body size (the most common proxy to status) may be influenced by demographic features, such as population density [21]. If only one of the male mating tactics involves fighting, for instance, it is expected that the relative costs of this tactic (compared to the alternative) will increase with population density, as encountering rival males becomes more likely. This is illustrated by male dimorphic bulb mites and soil mites, in which the average fitness of males from the fighter morph decreases with increasing numbers of rival males [22], and the probability that nymphs develop into the fighter morph diminishes with increasing population density [23]. During development, nymphs of these species are capable of assessing population density through a colony pheromone, and this information seems to influence the switchpoint that nymphs must reach in order to express the fighter phenotype as adults [24,25]. In male dimorphic insects, population density also seems to alter the selective pressure on each morph, driving the evolution of male dimorphic horns in dung beetles [26,27], and male dimorphic forceps in earwigs [28].

The only study that has directly assessed the shape of the functions that describe how fitness varies with body size for alternative male phenotypes was conducted with the dung beetle *Onthophagus taurus*[29], and corroborated the status-dependent selection model [11]. In this same species, other studies have suggested that an increase in the density of competing males results in a scenario in which the greater probability of encountering rival males would only allow the largest males to benefit from engaging in fights [26,30]. With this rationale comes the prediction that high population density would make the alternative tactic (sneaking by minor males) advantageous over a wider range of body sizes, moving the switchpoint for horn production to larger body sizes [26,27]. Correlational data on population density and horn allometry across several populations of *O. taurus* have supported this prediction [26,30]. A comparative analysis of 14 species of dung beetle also suggests that horns are less likely to evolve in species with greater mean crowding [31]. However, another prediction that naturally derives from this rationale is that, among majors, selection should favor steeper horn allometries under high densities. This is because the increased probability of encountering rivals under higher population densities should cause selection on long horns to be stronger, as horn length is known to be an important predictor of fight outcome [32]. Importantly, this new prediction, and the traditional prediction regarding the influence of population density on the evolution of switchpoints for horn production are not mutually excluding. In other words, higher population densities could result both in more minor males (due to larger switchpoint body sizes for horn production) and a steeper horn allometry among major males.

Thus, the development of juveniles in male dimorphic species should be sensitive to cues of the population density that will be faced by the adult. However, juveniles from holometabolous insects commonly develop in environments that are completely different from the environment in which they will face adult competition [33]. In fact, *Onthophagus taurus* larvae develop completely isolated from conspecifics inside enclosed brood masses, and are therefore probably unable to assess cues related to adult population density. On the other hand, parental individuals are in a position to perceive the environmental factors that will influence the future fitness of their developing offspring, and may adjust their provisioning decisions accordingly. Indeed, parental dung beetles provide offspring with all resources required for their development, which is in turn the primary determinant of adult phenotype, making male morph largely determined by parental provisioning decisions [34]. As a result, female *O. taurus* could in theory be capable of perceiving population density and when the population density is high, responding adaptively by producing more minor males in their offspring and/or producing major offspring with longer horns.

Here we tested this novel prediction by experimentally manipulating the social environment and the number of potential mates experienced by females of the dung beetle *O. taurus*, and later examining the effects of these maternal treatments on the phenotype of male offspring. The manipulation of social environment consisted on rearing females in isolation or with other females, which should affect the females’ perception of population density. The rationale behind manipulating the number of potential mates (in addition to social environment) was that females could theoretically assess the intensity of male-male competition that will be faced by their offspring through their perceived population density and/or through the number of males that they encountered and mated with. Our approach consisted of a laboratory experiment that standardized all other environmental factors (light cycle, temperature, humidity), allowing us to test directly the influence of perceived population density (social environment) and number of potential mates on the expression of alternative male tactics in the next generation. Moreover, we analyzed the response of females on two levels, namely the amount of dung they provided to their offspring, and the body size and horn allometry of their male offspring.

## Methods

### Collecting and rearing dung beetles

In January 2010, we collected adult *Onthophagus taurus* from cattle pastures in Margaret River, Western Australia. We maintained these individuals in the laboratory in single-sex populations for two weeks, with access to fresh cow dung *ad libitum*, in order to ensure that they were sexually mature. We then established male–female pairs in approximately 200 separate breeding chambers (PVC piping of 25 cm in length and 6 cm in diameter) that were three-quarters filled with moist sand and 250 ml of cow dung. After one week, we sieved these chambers to retrieve the brood masses, which were buried in moist sand and incubated at 25°C until adults emerged. This is a well established protocol for breeding *O. taurus*[29,34]; we repeated the procedure for two generations.

### Experimental groups in the pre-mating period

After emergence, second-generation offspring from the field-caught beetles were maintained in single-sex populations for two weeks, which corresponds to the pre-mating feeding period. We established these pre-mating populations in the same type of PVC piping used as breeding chambers, which were again three-quarters filled with moist sand and with dung provided *ad libitum*. Male chambers housed 20 males each (males were always reared in the same density), 12 of the minor morph and 8 of the major morph, which reflects the proportion (0.60) of minor males found in natural populations of *O. taurus* in Western Australia [35]. Meanwhile, female chambers were designated to one of two pre-mating experimental treatments that differed in social environment, and thus in the females’ perception of population density: the low-density group (L) housed only one female per chamber (n = 60 females); the high-density group (H) housed 20 females per chamber (n = 80 females in four different chambers). Natural populations of *O. taurus* vary in density from one to about 1,000 beetles per kilogram of dung [26]. Therefore, the densities we used here are well within the range of natural conditions to which the beetles might be adapted by natural selection.

### Experimental groups in the mating period

After the pre-mating period, sexually mature males, and females from both density groups, were randomly assigned to one of two mating experimental groups: in the singly-mated group (S), one minor male was paired to one female in a breeding chamber (n = 40 chambers); in the multiply-mated group (M), ten minor males were paired to ten females in each breeding chamber (n = 8 chambers). Therefore, sex ratio in all mating groups was 1:1. Only minor males were used in this stage of the experiment, because it has been shown that females of *O. taurus* provide more resources to their offspring when mated with males with large-horns [36], and we wanted to mitigate any paternal effects in our experiment, and focus only on the response of females to population density and number of mates. Males and females in these groups were kept together for five days, which is more than enough time for females to mate multiple times with either one minor male (S group), or potentially with ten minor males (M group). At this stage, pairs were not allowed to start building brood masses, as they were only provided with approximately 5 ml of cow dung to feed on, which is not enough to build brood masses. Females from the M group chambers in which more than one female died during the experiment were not used in the following stage of the experiment.

### Brood-provisioning period

After the mating period, we established females from all experimental groups individually in breeding chambers (as described above) with 250 ml of cow dung, in order to allow them to build brood masses and lay eggs. No males were included at this stage of the experiment, as we wanted females to provide their offspring alone, avoiding that any response of the females to the treatments were confounded with variation in males’ assistance to provisioning [34]. The sample size for this stage of the experiment was 20 females for each combination of experimental groups: high-density and singly-mated (HS); high-density and multiply-mated (HM); low-density and singly-mated (LS); and low-density and multiply-mated (LM). We did this by randomly selecting five out of ten females from each successful M group chamber (where no more than one female died) and using all females from the S group (all of them survived and produced offspring). We weighed (to 0.01 g, using a top pan balance) and measured (to 0.01 mm, using a digital caliper) these females for pronotum width just before establishing them in the breeding chambers, and checked that females from the different combinations of experimental groups (HS, HM, LS or LM) did not differ in either weight (*F*_3, 76_ = 1.201; *P* = 0.315, n = 80 females) or pronotum width (*F*_3, 76_ = 0.657; *P* = 0.581, n = 80 females).

### Assaying female responses to the treatments

After one week, we sieved breeding chambers and retrieved the brood masses produced by females of all experimental groups. We weighed each brood mass (to 0.01 g) using a top pan balance, buried them in moist sand inside individual plastic boxes (7 × 7 cm base, 5 cm height), and kept them at 25°C until emergence. We scored newly emerged adults for sex, and then froze them. For all male offspring, we next measured pronotum width (to 0.01 mm, using a digital caliper) and horn length. In order to measure horn length, we detached a beetle's head and mounted it on a pin under a *Leica* MZ75 dissecting microscope. We captured images on a *Leica* IC D digital camera and analyzed images using the free software *Image J*[37]. On each image, we measured horn length starting from the middle of the horn tip, following the horn curve until reaching the horizontal line level with the lowest point at the top of the head (measurement 4 in Figure 1 of [38]).

**Figure 1:**
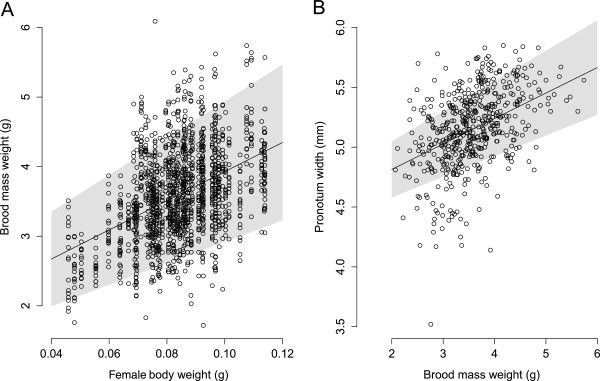
**The relationships between female weight, brood mass weight, and pronotum width of their male offspring. A.** The body weight of female *Onthophagus taurus* positively affected the weight of the brood masses they produced (the shaded area represent predicted values based on the 95% confidence intervals of the parameters of the model used). **B.** The weight of these brood masses positively affected the pronotum width of the male offspring that emerged from them (the shaded area represent predicted values based on the 95% confidence intervals of the parameters of the model used).

### Statistical analyses

In order to investigate whether females responded to the treatments by adjusting the amount of dung they provided to each offspring, we evaluated the effects of pre-mating group (L or H), mating group (S or M), female body weight, and all possible interactions on the weight of brood masses produced. We used the library 'nlme' [39] in R (version 2.14.0) to build linear mixed-effects models, considering the weight of brood masses produced as a response variable and female's body weight as a covariate. As a first step, we built three models with different sets of random effects: (1) no random effects; (2) female identity (to account for the fact that brood masses were not independent if they were built by the same female); and (3) the identity of the replicate mating chamber (to account for the fact that each female from the multiply-mated experimental group shared the same mating chamber with other females). In this third model, female identity was nested within the identity of the replicate mating chamber. We compared these models with log-likelihood tests, and determined that only female identity was a significant random effect, which we retained for the following step of the analysis. We used the same approach to select among different variance structures in our models, in order to fulfill the assumption of homogeneous variance of residuals across the predicted values of the models. In the second step of the analysis, we started with a null model and then added the fixed effects (pre-mating group, mating group, female body weight, and all possible interaction terms) sequentially, performing a log-likelihood ratio test at each step, to allow a quantification of the influence of each parameter. Importantly, because we were comparing models with different structures of fixed effects, we fitted the models by maximizing their log-likelihood (using the argument: method = "ML" in the function lme). Next, we repeated this analysis, but only considering the observations of brood masses from which adult offspring emerged successfully. These two analyses could return different results if the brood masses from which no offspring emerged were actually unfinished by the females. Similarly, we repeated this analysis again, but only considering the observations of brood mass weight for brood masses from which male offspring emerged. This analysis should only differ from the previous ones if brood mass provisioning by females was responding to the treatments in different ways, depending on the sex of the offspring being produced.

We used the same approach to analyze the pronotum width of the male offspring from our experimental groups. However, this time we added to the models the weight of the brood mass (instead of the female's body weight) and all interaction terms with the other predictor variables. Moreover, here, the random effect of the replicate mating chamber was important, so all models had this random effect in addition to the random effect of female identity (nested within the identity of the replicate mating chamber).

Regarding the effects of the treatments on the horn length of the offspring, we followed the steps proposed by [40] for the analysis of non-linear allometries. Firstly, we pooled the offspring from all experimental groups, plotted a log-log scatterplot of horn length on pronotum width in which ΔX = ΔY (see [41] for why this is important), and concluded that there was a clear continuous relationship that was not a straight line. Importantly, we added one to the natural values of horn length before log transformation, in order to avoid zeros (due to no horns in the smallest minor males) that would result in minus infinity after transformation. Next, we selected sigmoidal nonlinear models that are appropriate to explain biological growth curves, and used the function 'nls' and the library 'grofit' [42] in R (version 2.14.0) to fit these models (three-parameter logistic, four-parameter logistic, Weibull growth curve, and Richards’ growth function; see Chapter 20 in [43]) and a simple linear regression to the data. Finally, we compared all these models on the basis of the Akaike information criterion (AIC), calculated as [2 × (− log likelihood) + 2 × (the number of parameters in the model)].

After finding the nonlinear model that best fitted the horn allometry of *O. taurus*, we again used log-likelihood tests to select among different variance structures that would fulfill the assumption of homogeneous variance of residuals across the predicted values of the model. We then built a set of models in which one (or all) of the parameters of the nonlinear model were allowed to have different values for each pre-mating or mating experimental groups, and used log-likelihood tests to compare these models with a model in which the parameters had a single value for all experimental groups. This approach allowed us to infer if the horns of the offspring from the different experimental groups differed in any of the parameters that describe their sigmoidal allometry. An advantage of this approach is that some of the parameters tested have specific and straightforward biological interpretations (see Discussion).

## Results

During the provisioning period of one week, females produced on average 19.2 (SD = 3.2, range = 7–27, n = 80 females) brood masses each, and the combined weight of brood masses produced by each female averaged 68.95 mg (SD = 14.77 mg, range 24.58 - 99.19 mg, n = 1,537 brood masses). The body weight of females positively affected the weight of individual brood masses they produced (Figure 1A), but there was no effect of pre-mating group (L or H), mating group (S or M) nor any interaction involving these variables on the weight of brood masses (Table 1). The results of this analysis do not change significantly if we only consider the observations of brood masses from which male offspring or any offspring emerged successfully (analyses not shown). The weight of the brood masses affected the pronotum widths of the male offspring that emerged from them (Figure 1B), but there was again no effect of pre-mating group (L or H), mating group (S or M) or any interaction involving these variables on this trait (Table 2).

**Table 1:** **Model selection statistics for the weight of brood masses produced by females of *Onthophagus taurus***

**Model**			**Pairwise sequential model comparisons**	
**Fixed effects**	***df***	**log Likelihood**	**Likelihood ratio**	***P-*value**
Null	4	- 1153.7		
Female weight	5	- 1133.3	40.81	**< 0.0001**
Female weight + M group	6	- 1132.3	2.14	0.14
Female weight + PM group + M group	7	- 1132.1	0.27	0.60
Female weight + PM group + M group +	8	- 1131.6	1.07	0.30
Female weight : PM group				
Female weight + PM group + M group +	9	- 1131.5	0.10	0.75
Female weight : PM group +				
Female weight : M group				
Female weight + PM group + M group +	10	- 1131.5	0.01	0.91
Female weight : PM group +				
Female weight : M group +				
PM group : M group				
Female weight + PM group + M group +	11	- 1131.3	0.47	0.50
Female weight : PM group +				
Female weight : M group +				
PM group : M group +				
Female weight : PM group : M group				

Females were assigned to experimental groups that differed in population density during the pre-mating period (PM), and in the number of possible mates during the mating period (M). The effect of female weight was added as a covariate, female identity was added as a random effect in all models (estimated as 0.323 standard deviation in the full model), and the variance was modeled as a power function of the fitted vales with an estimated parameter value of 1.260 in the full model. Likelihood ratios were calculated as the absolute difference between the - 2 x log Likelihood of the two models being compared, and each model is being compared to the model that is one row above. Comparing these models in terms of their AIC values returns qualitatively similar results.

**Table 2:** **Model selection statistics for the pronotum width of male offspring from females of *Onthophagus taurus***

**Model**			**Pairwise sequential model comparisons**	
**Fixed effects**	***df***	**log Likelihood**	**Likelihood ratio**	***P*-value**
Null	5	- 114.19		
Brood mass weight	6	- 57.72	112.93	**< 0.0001**
Brood mass weight + M group	7	- 56.10	3.24	0.07
Brood mass weight + M group + PM group	8	- 56.18	0.16	0.69
Brood mass weight + M group + PM group + Brood mass weight : PM group	9	- 54.36	3.65	0.06
Brood mass weight + M group + PM group +	10	- 53.14	2.43	0.12
Brood mass weight : PM group +				
Brood mass weight : M group				
Brood mass weight + M group + PM group + Brood mass weight : PM group +	11	- 53.13	0.03	0.87
Brood mass weight : M group +				
PM group : M group				
Brood mass weight + M group + PM group +	12	- 53.08	0.09	0.76
Brood mass weight : PM group +				
Brood mass weight : M group +				
PM group : M group +				
Brood mass weight : PM group : M group				

Females were assigned to experimental groups that differed in population density during the pre-mating period (PM), and in the number of possible mates during the mating period (M). The effect of brood mass weight was added as a covariate, the replicate mating chamber was added as a random effect, and female identity (nested within the replicate mating chamber) was also added as a random effect in all models. The random effects in the full model were 0.069 standard deviation for the replicate mating chamber and 0.115 standard deviation for female identity. The variance was modeled as a power function of the fitted values with an estimated parameter value of - 4.480 in the full model. Likelihood ratios were calculated as the absolute difference between the - 2 x log Likelihood of the two models being compared, and each model is being compared to the model that is one row above. Comparing these models in terms of their AIC values returns qualitatively similar results.

**Figure 2:**
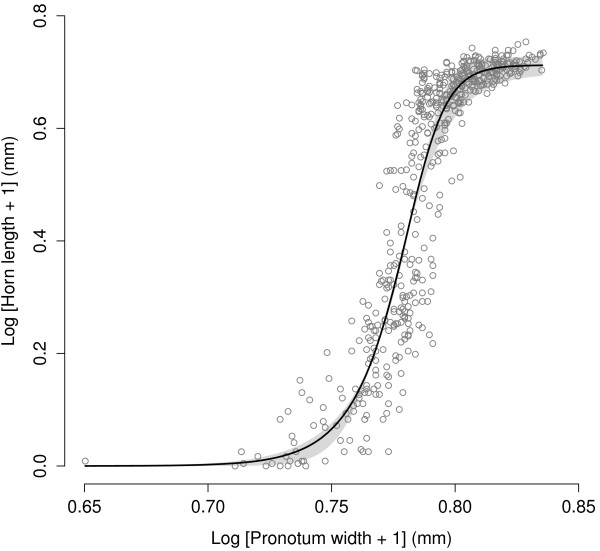
**The allometry of horn length on pronotum width of males in *Onthophagus taurus.*** Males in the sample were the pooled offspring produced by females from all our experimental groups (see Methods). Both axes were transformed using natural logarithms, the curve was fitted with a Richards’ growth function (parameters in Table 3), and the shaded area represents predicted values based on the 95% confidence intervals of the parameters of the model used.

**Figure 3:**
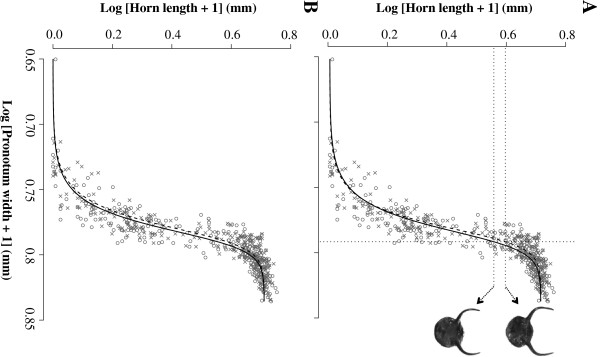
**The horn length allometry of the male offspring produced by females from different experimental groups.** These groups differed in the population density experienced by females during their pre-mating (PM) period. Offspring produced by females from the low-density experimental group are indicated by open circles and a continuous curve, whereas offspring produced by females from the high-density experimental group are indicated by crosses, and a broken curve. Again, both axes were transformed using natural logarithms, and the curves were fitted with a Richards’ growth function, but this time (**A)** only parameter μ (Model 4), (**B)** or parameter λ (Model 5) were allowed to vary between the experimental groups, whereas parameters A and *v* were always common across experimental groups (see Table 4). **A.** According to the model that allows only parameter μ to vary between experimental groups (Model 4, Table 4), the vertical dotted line depicts a value of body size for which the model predicts a horn length 8.4% greater for offspring of females that experienced high population density in their pre-mating period (upper dotted horizontal line) than for offspring of females that experienced low population density during their pre-mating period (lower dotted horizontal line). This line was chosen to depict the biggest possible difference in the horns of offspring produced by females of the two experimental groups described above.

Regarding horn length, the model that best described the allometry between this trait and the pronotum width of males was the sigmoidal Richards’ growth function (Table 3, Figure 2). Interestingly, at least three parameters of the Richards’ growth function have biological interpretations: parameter A is an asymptote, indicating the asymptote for horn length; parameter μ is the maximum slope of the curve, indicating the biggest increase of horn length with body size; *λ* parameter is a lag-phase_,_ indicating the body size after which significant horn growth occurs, and can be interpreted as the switchpoint between male morphs. Parameter *v* is a shape parameter that affects how close to the asymptote maximum growth occurs, regardless of the lag-phase, but the biological interpretation of this parameter is less straightforward. Moreover, the model that allowed all of the parameters of the Richards’ growth function to have different values for the L and H pre-mating groups (Model 2, Table 4) was significantly better than the model that had the same parameters across both pre-mating groups (Model 1, Table 4). When looking at each specific parameter, the models that allowed only μ or only *λ* to vary between the L and H pre-mating groups (Models 4 and 5, Table 4, Figure 3) were also significantly better than the model that had the same parameters across both pre-mating groups (Model 1, Table 4). On the other hand, the model that allowed all of the parameters of the Richards’ growth function to have different values for the S and M mating groups (Model 7, Table 4) was not significantly different from the model that had the same parameters across both mating groups (Model 1, Table 4). Therefore we concluded that Models 4 and 5, where parameters μ or *λ* vary between the pre-mating groups, are the minimal adequate models that fit the data best.

**Table 3:** **Models fitted to the allometry between horn length and pronotum width of male *Onthophagus taurus***

**Model**	**AIC**	Δ** AIC**	**Formula and parameters**
**Sigmoidal Richards’ growth function**	**- 983.889**	**0**	$Y(x)=A{\left[1+v\times exp(1+v)\times exp(\mu \;{\left(1+v\right)}^{1+1/v}\times (\lambda -\times )/A)\right]}^{\left(-1/v\right)}$
			**A (asymptote) = 0.70836**
			**μ (maximum slope) = 18.52390**
			*λ ***(lag-phase) = 0.75852**
			***v *(shape parameter) 2.70687**
Sigmoidal Weibull growth function	- 981.318	2.571	$Y(x)=A-Drop\times exp(-exp(lrc)\times x^{\mathrm{pwr}})$
			A (asymptote) = 0. 700975
			Drop (asymptote minus y intercept) = 0. 684813
			lrc (ln rate constant) = 13. 622203
			pwr (power x is raised to) = 55. 655926
Four-parameter logistic	- 981.127	2.762	Y(x)=lA+(μA-lA)/(1+exp((xmid-×)/scal))
			lA (lower asymptote) = 0. 0447065
			uA (upper asymptote) = 0. 7220289
			xmid (x value for inflection point) = 0. 7787523
			scal (scale parameter) = 0. 0091249
Three-parameter logistic	- 977.034	6.855	Y(x)=A/1+exp((xmid-x)/scal)
			A (asymptote) = 0.7306256
			xmid (x value for inflection point) = 0.7774247
			scal (scale parameter) = 0.0102605
Linear	- 721.143	262.746	$Y\left(x\right)=a+bx$
			a (intercept) = −6.3043
			b (slope) = 8.6193

Male offspring were pooled across females from all experimental treatments. The best model is in bold and the remaining models are sorted below by increasing values of AIC.

**Table 4:** **Model selection statistics for the allometry between horn length and pronotum width in *Onthophagus taurus***

	**Model**			**Pairwise model comparisons**	
	**Parameters as functions of experimental groups**	***df***	**log Likelihood**	**Likelihood ratio**	***P***-**value**
*1*	*A, μ, λ*, and *v common across groups*	*6*	*524.65*		
	Pre-mating group (PM)				
2	All parameters ~ PM	10	533.39	17.47	**0.002**
3	A ~ PM; μ, *λ*, and *v* common across groups	7	524.68	0.06	0.81
4	μ ~ PM; A, *λ*, and *v* common across groups	7	533.20	17.11	**< 0.0001**
5	*λ* ~ PM; A, μ, and *v* common across groups	7	533.04	16.78	**< 0.0001**
6	*v* ~ PM group; A, μ, *v*and *λ* common across groups	7	525.49	1.68	0.20
	Mating group (M)				
7	All parameters ~ M	10	525.10	0.90	0.92
8	A ~ M; μ, *λ*, and *v* common across groups	7	524.75	0.21	0.65
9	μ ~ M; A, *λ*, and *v* common across groups	7	525.05	0.81	0.37
10	λ~ M; A, μ, and *v* common across groups	7	524.95	0.60	0.44
11	*v* ~ M; A, μ, and *λ* common across groups	7	524.79	0.29	0.59

All models were fitted using the Richards’ growth function (see Table 3). Individuals in the sample are the male offspring produced by females from experimental groups that differed in population density during the pre-mating period (PM), and in the number of possible mates during the mating period (M). The set of models being compared is composed of a full model with common parameters across experimental groups (Model 1), and models in which one (or all) of the parameters were allowed to have different values for each pre-mating or mating experimental groups (indicated by ~ PM or ~ M). The variance was modeled as an exponential function of the fitted values with an estimated parameter value of - 1.853 in the full model with common parameters across experimental groups (Model 1). Likelihood ratios were calculated as the absolute difference between the - 2 x log Likelihood of the two models being compared, and every model is being compared to the model in italic (Model 1). Comparing these models in terms of their AIC values returns qualitatively similar results.

It was not possible to model the random effect of female identity in the models describing the horn allometry of *O. taurus*. The reason for this is that nine of the 80 females used in the experiment produced fewer than four male offspring. Therefore, it is impossible to estimate the effect of each female on all four parameters of the Richards’ growth function. However, to make sure that our results were robust when accounting for the number of females used, we extracted the horn length residuals from the Richards’ growth function model that had the same parameters across experimental groups (Model 1, Table 4). Next, we averaged the residual horn length for the male offspring of each female, and compared these averages across experimental groups using Welch's t-tests for samples of unequal variances. This approach is very conservative and avoids any possible pseudo-replication due to the non-independence among offspring from the same female. Regarding the pre-mating experimental groups, the averages of son's residual horn length per female were higher in the high-density group than in the low-density group (t_66.521_ = −2.38, *P* = 0.02). Regarding the mating experimental groups, the averages of son's residual horn length per female were not different between the singly-mated and the multiply-mated groups (t_75.455_ = −0.60, *P* = 0.55).

## Discussion

Theory predicts that variations in dung beetle population density should have different fitness consequences for each male morph, so that higher population density selects for a higher frequency of minors [26,27,30] and/or greater expenditure on weaponry in majors. Moreover, given that female dung beetles provide their offspring with all the resources they need to reach adulthood [34], non-genetic paternal (e.g. size; [36]) and maternal (e.g. size; [34]) effects influence the morph adopted by male offspring. Following this rationale, in the present study we predicted that female *O. taurus* would be capable of responding to population density by changing the phenotype of their offspring. Our results suggest that beyond the maternal effects that arise from size variation – a seemingly adaptive effect is present whereby mothers who experienced high population density produce major offspring with longer horns. Below we build on this finding and their theoretical implications.

### More minors or better majors as a response to population density?

The model that best described the horn allometry of male offspring in this study was the four-parameter Richards’ growth function, which performed better than a linear model, a three-parameter logistic, a four-parameter logistic, and a Weibull growth curve (Table 3). Moreover, allowing two parameters (only μ or only *λ*) of the Richard’s growth function to vary between the low and high density pre-mating groups resulted in significantly better fit than a model that had the same parameters across both pre-mating groups (Table 4). Because the high density pre-mating group had a significantly higher value for parameter μ, the biological interpretation is that offspring from females in this group had a greater maximum increase of horn length with body size, i.e., a steeper horn length allometry (Figure 3A). Alternatively, because the high density pre-mating group also had a significantly lower value of parameter *λ*, another possible biological interpretation is that offspring of females in this group had a switchpoint for morph expression at smaller values of body size (Figure 3B). Both these interpretations have the same effect for major males: major offspring of females from the high density pre-mating group had longer horns across a wide range of body sizes than major offspring of females from the low density pre-mating group (Figure 3). However, for minor males, differences in parameters μ and *λ* have different implications. If only parameter μ is different across the pre-mating groups, minors of both groups are not different, as this parameter only affects the steepness of the curve after the switchpoint (Figure 3A). Meanwhile, if only parameter *λ* is different across the pre-mating groups, the switchpoint is moved to lower values of body size under high maternal density conditions, and minors are produced over a narrower range of body sizes (Figure 3B), i.e. there would be fewer minors.

Our original prediction was that females who experienced higher population densities could respond adaptively by producing a steeper horn allometry among major males and/or more minor males (due to larger switchpoint body sizes for horn production). Our results corroborate part of this hypothesis, consistently pointing towards longer horns in major males produced under the condition of high population density. This is because the two parameters of the Richards’ growth function that are significantly different between the pre-mating density groups have this same effect on major males (Figure 3). On the other hand, our results are inconclusive regarding the effect of pre-mating population density on minor males, as one of the parameters of the Richards’ growth function that is significantly different between pre-mating density groups has no implications for the frequency of minor males produced (Figure 3A). If anything, females actually produced fewer minor males under high density conditions, and not more, as we originally predicted (Figure 3B). This result challenges the prediction that more minor males should be produced under high population density, suggesting that minor males might actually have fewer mating opportunities than major males under high population densities. Nevertheless, this result should be interpreted with care, as it was not consistently supported by the two models that best fitted our data (Models 4 and 5, Table 4), whereas the horn length change in major males was.

The uncertainty regarding the effects of our experimental treatments on minor males was probably because we had fewer minor males than expected in the offspring of females from all experimental groups, when compared to the proportion of approximately 60% minor males commonly found in natural populations of *O. taurus* in Western Australia [35]. This occurred because we did not limit the reproductive activity of females by dung availability. In other words, we provided enough dung for females from all groups to produce more major offspring than they normally do in field conditions. Therefore, any differences detected in the proportion of major and minor offspring produced by females from different experimental groups could reflect adaptive allocation strategies to produce offspring that are better capable to cope with the population density that they will experience, isolating effects of resource limitation for females.

In conclusion, it has been argued that an increase in the density of competing males of *O. taurus* results in a greater probability of encountering rival males, which allows only the largest males to benefit from fights [27,44]. Earlier studies used this rationale to predict that higher population densities make the sneaking tactic of minor males advantageous over a wider range of body sizes, moving the switchpoint for horn production to larger body sizes [26,27], and predicted no change in the horn allometry of major males. However, our results do not support this prediction, showing that when females experience high population densities the switchpoint is either unchanged, or moved to smaller body sizes through maternal effects. In contrast, maternal effects clearly affect the horn allometry of major males, increasing the horn length of majors produced by females experiencing high population density. Even small differences in horn length can provide considerable advantages in male contests in this [32] and other dung beetle species [45,46], and horn length in *O. taurus* has strong independent effects on male competitive fitness [29]. Females thus appear able to use cues of population density to prepare their major sons for an environment in which competition will be intense.

### A new mechanism of maternal effect in *O. taurus*

Our results indicate that females of the dung beetle *Onthophagus taurus* are capable of perceiving population density, and responding by changing the horn allometry of the male offspring they produce. Maternal effects on male dimorphism are well known in dung beetles, because male morph is mainly determined by the quality and quantity of dung ingested by larvae [47,48], which in turn is collected and provisioned by the parental individuals [49]. As a consequence, parental effects through offspring provisioning are chief determinants of the alternative phenotypes adopted by dung beetles [34,36]. However, the females in our experimental treatments did not respond by changing their brood mass provisioning decisions, as we could not detect any effect of the experimental manipulations on the weight of the brood masses produced. Likewise, the body size (measured as pronotum width) of male offspring was also unaffected by our experimental manipulations. Rather, our data suggest that females seem capable of specifically influencing the horn growth of their male offspring. We suggest that the specific biological mechanism through which this maternal effect operates may be a transgenerational effect that goes beyond a maternal influence on offspring condition or body size *per se*.

The first hypothesis that we put forward is that the maternal effect we detected may be transmitted through the eggs laid by females. Maternal effects operating through egg size are not uncommon (references in [50]), and have been reported in male dimorphic mites [51]. But a straightforward effect of egg size on offspring morph would probably affect offspring body size as well (as in [51]), which was not the case in our study. Alternatively, egg composition could in theory affect offspring horn growth without a detectable effect on offspring body size. Egg composition is known to affect offspring phenotype in birds [52] and fish [53]. In the seed beetle *Stator limbatus,* females change the size and probably the composition of their eggs according to the host plant they encounter while maturing eggs, and the survivorship of their offspring is greatly affected by this plasticity in egg production [54]. This kind of maternal effect through egg composition might also be present in male dimorphic fig wasps, in which females probably produce chemicals that influence male morph determination in the offspring by acting on the genetic cascades of their genomes [55]. Not much is known about egg composition in dung beetles, but we suggest that this is a promising topic that could reveal hidden forms of maternal effects on the male offspring of these animals.

Another possibility is that the maternal effect detected in this study is transmitted through a feature of the brood masses that could not be detected in our experiment, such as the composition of the saliva components used by females in the process of building brood masses. The naturalist Jean Henri Fabre [56] was the first to notice that the inner walls of brood masses built by females of *Onthophagus* dung beetles are coated with a shiny and greenish semifluid, which he suggested to be produced by the mother, probably as the result of semi-digested food. It is possible that this substance contains hormones derived from the saliva of females, which can have an effect on the development of offspring horns. By altering the hormone content of their saliva and consequently of the processed dung provided for their young, females could influence horn development of their offspring through what would be a new mechanism for maternal effects in these animals.

Both a maternal adjustment to the composition of the eggs or to the composition of the brood masses could cause major phenotypic effects in their offspring, potentially through epigenetic effects, such as DNA methylation [57]. Epigenetics seem to play a major role in the regulation of phenotypic plasticity [58], especially for insects that present conditional dimorphisms [59,60], such as the well-studied plastic expression of worker and queen castes in the honeybee [61,62].

## Conclusion

As with any kind of phenotypic plasticity, male dimorphism that results from conditional strategies has an underlying genetic variation, but the adult phenotype adopted by each male is mainly determined by environmental conditions [18]. Therefore, it may seem that selection can only target conditional strategies at two levels: the genetic switchpoint that determines which alternative phenotype will be expressed in each environmental condition [19]; and the physiological cascade that is involved in phenotypic expression *per se* (i.e., the downstream mechanisms, [63]). However, if maternal effects play a role in offspring’s morph determination (e.g. [34,64]), then the environments experienced by developing juveniles can also evolve over generations. This is because although maternal effects are environmentally transmitted, the genes that affect their expression (which reside in the mothers’ genomes) are subject to selection.

To date, examples of maternal effects on male dimorphism come from a few species of fish [65], fig wasps [55], burrowing bees [64], and also dung beetles [34,36]. These maternal effects are expected to evolve whenever females are better capable of assessing the environmental conditions that their offspring will experience than the offspring themselves [3]. Our study describes a new type of maternal effect in dung beetles, which is likely to operate through egg or brood mass composition. This maternal effect probably allows females to respond to population density adaptively, preparing at least their major offspring for the level of sexual competition they will face as adults. In Figure 3 it can be visualized that major offspring of the same body size can have horns up to 8.4% longer if they were produced by females that experienced high density than if they were produced by females that experienced low density. This difference is enough to significantly increase the chances of the major male with longer horns to win a fight [32]. Finally, this maternal effect represents a novel transgenerational response of alternative reproductive tactics to population density.

## Abbreviations

H: High-density experimental group; L: Low-density experimental group; M: multiply-mated experimental group; S: singly-mated experimental group; PM: Pre-mating period; M: Mating period; AIC: Akaike information criterion.

## Competing interests

The authors declare that they have no competing interests.

## Authors’ contributions

BAB carried out the experiment, analysed the data and drafted the manuscript. JLT and LWS participated in conceiving the study, designing the experiment, and guiding and reviewing the writing of the manuscript. All authors approved the final version of the manuscript.
